# Ferroptosis Signaling in Pancreatic β-Cells: Novel Insights & Therapeutic Targeting

**DOI:** 10.3390/ijms232213679

**Published:** 2022-11-08

**Authors:** Suma Elumalai, Udayakumar Karunakaran, Jun-Sung Moon, Kyu-Chang Won

**Affiliations:** 1 Innovative Center for Aging Research, Yeungnam University Medical Center, Daegu 42415, Korea; 2 Department of Internal Medicine, College of Medicine, Yeungnam University, Daegu 42415, Korea

**Keywords:** iron, NADPH oxidase, oxidative stress, diabetes, reactive oxygen species, ferroptosis

## Abstract

Metabolic stress impairs pancreatic β-cell survival and function in diabetes. Although the pathophysiology of metabolic stress is complex, aberrant tissue damage and β-cell death are brought on by an imbalance in redox equilibrium due to insufficient levels of endogenous antioxidant expression in β-cells. The vulnerability of β-cells to oxidative damage caused by iron accumulation has been linked to contributory β-cell ferroptotic-like malfunction under diabetogenic settings. Here, we take into account recent findings on how iron metabolism contributes to the deregulation of the redox response in diabetic conditions as well as the ferroptotic-like malfunction in the pancreatic β-cells, which may offer insights for deciphering the pathomechanisms and formulating plans for the treatment or prevention of metabolic stress brought on by β-cell failure.

## 1. Introduction

Despite considerable advances in treatment, the prevalence of diabetes mellitus (DM) is increasing worldwide, endangering human health and placing an economic burden on society. Diabetes mellitus is a diverse condition caused by progressively impaired insulin production from β-cells and insulin resistance in target tissues. There are several types of diabetes, of which type I and type II are the most common. The dysfunction and destruction of pancreatic β-cells in type 1 diabetes (T1D) are caused by cytotoxic T cells and pro-inflammatory cytokines. Obesity, hyperglycemia, peripheral insulin resistance, and cytokine levels are the main features of type 2 diabetes (T2D), a complicated metabolic condition that results in a relative lack of insulin and β-cell failure. Apoptosis is the endpoint of β-cell death in both forms of disease [1,2,3,4]. Knowledge of the pathophysiology of this disease has now entered a new era based on an understanding of the biology and critical reappraisal of the pathobiology of β-cell failure. Increasing evidence suggests that iron accumulation is linked to an elevated risk of both type 1 and type 2 diabetes and is proposed to be involved in the pathophysiological mechanisms of β-cell failure [5,6,7,8]. This suggests that ferroptosis, an iron-dependent and non-apoptotic cell death, is one of the triggering events in β-cell pathophysiology and is characterized by the accumulation of toxic lipid reactive oxygen species (ROS). The key initiating step of ferroptosis is the inhibition of cystine uptake into cells, which can be prevented by iron chelation [9]. Many physiological and pathological factors perturb iron function and induce metabolic diseases, depending on the duration and degree of metabolic stress. In this review, we describe the critical events involved in ferroptosis with respect to their relevance to β-cell failure in diabetes.

## 2. Involvement of Iron and β-Cell Function: An Overview

The second-most prevalent metal in the Earth’s crust is iron, which is also a vital micronutrient for life. Its biological significance is demonstrated by the high prevalence of human diseases caused by disturbances in iron homeostasis. Iron is a transition metal that can adopt various oxidation states. The ferrous (Fe^2+^) and ferric (Fe^3+^) forms are the most prevalent, and this transition is largely responsible for the biological significance of iron. Iron is a crucial cofactor for electron transfer because of its propensity to receive and provide electrons, and its adaptable coordination chemistry is crucial for its versatility in binding to biological ligands [10]. Because of its chemical makeup and potential for harm, cells have created a sophisticated system for handling iron to maintain it at adequate and safe levels. Carriers and receptors bind and transport ions across membranes, and enzymes and buffering proteins regulate their redox state and free level. Buffering proteins also act as protective buffers. Iron-binding protein expression is regulated by iron regulatory proteins, according to ion density. Several of these proteins are present in pancreatic β-cells, such as the transferrin receptor (TrfR), the mitochondrial iron-storage protein frataxin, the cytosolic iron-storage proteins ferritin H and L chains, the iron-export regulatory hormone hepcidin (which is found in the insulin granules), the iron chaperone lipocalin 2, and the v-ATPase, supporting the hypothesis that β-cells have a classical iron metabolism [11,12,13,14,15,16]. After binding to TrfR, transferrin-bound iron is taken up in the blood. After that, the metalloreductase six transmembrane epithelial antigen of prostate family member 3 and DMT1 endocytose the transferrin-TrfR complex (STEAP 3). Iron is liberated from transferrin inside the endosome and reduced via a drop in pH by metalloreductase STEAP 3 and proton pump v-ATPase. Subsequently, the iron is delivered to the cytoplasmic labile iron pool (LIP) via DMT1 over a v-ATPase-provided proton gradient. Cytosolic LIP receives iron in the form of ferrous iron, which is then transported to ferritin for storage or to the nucleus, ER, mitochondria, and Fe-S proteins for functional use by iron chaperones such as lipocalin 2 and poly (rC) binding protein (PCBP) 1 and 2 [17]. Ferritin-bound iron is stored in insulin granules or adjacent to the plasma membrane in β-cells, and iron is exported from β-cells through ferroportin, the activity of which is suppressed by paracrine or autocrine effects of the small peptide hormone hepcidin [11,14,18]. Hepcidin binding to FPN induces its internalization and lysosomal degradation, thus directly inhibiting iron release into circulation from the sites of iron absorption, recycling, and storage. Furthermore, β-cells release hepcidin in response to glucose stimulation, indicating that ferroportin binding inhibits iron export as a positive feedback mechanism in iron management during glucose-stimulated insulin production [14]. Hence, due to its dual nature, iron levels must be maintained within a tight physiological range to avoid the detrimental consequences of both iron deficiency and excess iron.

**Figure 1:**
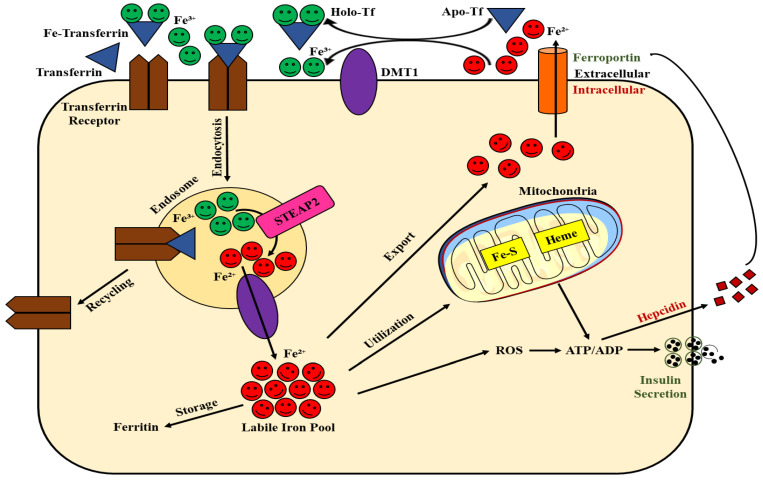
Role of iron in β-cell physiology.

Although iron has been detected in almost all intracellular organelles, given the significance of the mitochondria and ER in β-cell function and dysfunction as well as the therapeutic implications that follow, understanding the metabolism of iron in β-cell mitochondria and ER is of special interest. Most of the labile iron pool, in contrast, is transported to the mitochondria, where it combines with heme and Fe-S clusters. Iron is exported from endosomes by DMT1; however, it is unclear how iron travels from the cytosol to the inner mitochondrial membrane. A wide body of evidence suggests a possible role of frataxin, an iron chaperone located in the mitochondrial matrix that was observed to interact with the Fe-S-cluster assembly and presumably appears to be a key activator of mitochondrial energy flux by oxidative phosphorylation [19,20,21]. In this way, frataxin acts as a coordinator of the electron transport chain, leading to increased mitochondrial membrane potential Δψ_m_ and elevated cellular ATP content. However, disruption of the frataxin gene, specifically in pancreatic β-cells, leads to a reduction in insulin-secretory capacity and impaired glucose tolerance, resulting in overt DM due to a loss of β-cell mass. Furthermore, disruption of frataxin leads to increased levels of ROS within pancreatic islets, which in turn are associated with increased apoptosis and decreased proliferation [22]. This finding can be interpreted in two ways: first, it is necessary for complex II to properly utilize electrons. Ubiquinone (Q) is not entirely reduced to ubiquinol (QH2), and an excess of the intermediate semiquinone form results from improper electron incorporation into the respiratory chain. By interacting with molecular oxygen to produce superoxide radicals and induce oxidative stress in the mitochondria, the formation of this radical semiquinone has been linked to a pro-oxidizer impact. Second, frataxin disruption may decrease mitochondrial ATP production, which leads to reduced insulin exocytosis and secretion. In addition, frataxin deficiency can exacerbate ER stress in β-cells [23]. Therefore, it is of great interest to understand how the disruption of frataxin contributes to β-cell death and warrants further study (Figure 1).

## 3. Role of Iron Accumulation and β-Cell Dysfunction

Hereditary hemochromatosis is a common genetic disorder of iron metabolism where iron accumulates specifically in the endocrine pancreas resulting in decreased insulin secretion and increased protein oxidation and beta cell apoptosis [6,24]. Furthermore, experimental studies have indicated that patients with transfusional iron overload have increased iron deposition in β-cells, which may result in hyperglycemia and DM [25,26]. Paradoxically, iron is also deposited in the muscles and livers of patients with hemochromatosis, causing decreased glucose uptake and insulin resistance. In addition, the exact mechanisms by which iron deposition occurs are not known.

Consistent with the ability of iron to readily accept and donate electrons, iron is an essential cofactor for electron transfer, and its flexible coordination chemistry is key to its versatility in binding to biological ligands. Paradoxically, the same chemical properties that render iron biologically essential also underlie the toxicity of excess iron. In eukaryotic cells, small concentrations of labile Fe^2+^ are found in the cytosol and mitochondrial matrix; the lysosome also has redox-active iron derived from extracellular sources, and these cells also have the ability to break down ferritin and iron-rich intracellular organelles, such as mitochondria [27,28]. These redox-active iron pools can directly catalyze the creation of harmful free radicals using Fenton chemistry [29]. Both iron-dependent ROS-producing enzymes and labile iron are thought to contribute to ROS-dependent cell damage and cell death. Because of the difficulties in defining the targets and effects of ROS that are significant to mortality as well as the consequences of iron accumulation on cell function, this phenomenon is currently the subject of active research.

## 4. Is Ferroptosis the Result of Iron Accumulation and β-Cell Dysfunction?

Pancreatic β-cells are characterized by a relatively high iron content and a dependence on mitochondrial respiration for insulin secretion, which has long been considered an argument that oxidative stress is highly relevant in pancreatic β-cell dysfunction. Moreover, iron has long been recognized as a signaling molecule in the inflammatory response to the induction of insulin resistance, both in vitro and in vivo [30,31]. Signaling pathways that affect iron metabolism have also been shown to modulate ferroptosis. Thus, iron-dependent cell death may be especially important in DM. Metabolic dysfunction has been explained by several theories, including mitochondrial dysfunction, oxidative stress, ER stress, hyperglycemia (glucotoxicity), dyslipidemia, and the concomitant presence of both hyperglycemia and dyslipidemia (glucolipotoxicity) [32]. Another mechanism by which this environment is conducive to the development and/or progression of diabetes is the activation of chronic inflammation.

### 4.1. The Role of DMT1 in Ferroptotic Signaling

Indeed, experimental evidence has shown that the pro-inflammatory cytokine IL-1β induces divalent metal transporter 1 (DMT1) expression, which correlates with increased β-cell iron content and ROS production via an increased intracellular LIP. This was associated with elevated levels of the iron import mediators lipocalin-2 (Lcn2) and TrfR and decreased levels of ferroportin, an iron exporter. Iron chelation or genetic knockdown of DMT1 reduced cytokine-induced ROS formation and cell death. Interestingly, glucose-stimulated insulin secretion in the absence of cytokines in DMT1 knockout islets was defective, highlighting the physiological role of iron and ROS in the regulation of insulin secretion [33]. Furthermore, there is considerable evidence suggesting that the expression of DMT1 and LCN2 is induced by pro-inflammatory cytokines in pancreatic β-cells [34,35]. Whether this increase in LCN2 levels is a cause or result of metabolic dysregulation and whether it has an impact on disease progression have not been examined. Moreover, LCN2 is a neutrophil gelatinase-associated protein that influences iron homeostasis by forming a ternary complex with a siderophore as its cofactor, and it serves as a defense mechanism of the innate immune response system [36,37]. In addition to restricting iron availability, LCN2 also exerts a cytoprotective effect against STZ in a short-term HFD mouse model of diabetes by improving β-cell mass and promoting β-cell proliferation [38]. However, this also suggests that future studies are needed to understand the possible interference of LCN2 in the pathophysiology of pancreatic β-cell iron dysregulation. It has also been shown that by increasing NF-kB transcriptional activity, abnormal cytokine-dependent increases in cellular iron import via DMT1 primes β-cells for ROS-mediated inflammatory damage [33]. There is evidence that NF-kB controls the promoter activity of a number of genes whose expression has changed as a result of cytokine exposure, which is implicated in the deleterious effects of β-cells [39,40,41,42]. Indeed, it has been observed that pro-inflammatory cytokines upregulate the activation of inducible nitric oxide synthase (iNOS) gene expression and the subsequent formation of NO, which, in part, leads to the loss of function and activation of oxidative stress linked to β-cell failure [43,44,45]. Importantly, peroxynitrite, a novel and reactive peroxide resulting from the rapid interaction of superoxide radicals with NO, mediates cytokine-induced damage [46,47]. Furthermore, evidence supports a key role of peroxynitrite in the Fe-S cluster of IRP1 destabilization, resulting in the inactivation of aconitase activity and inhibition of the Fe-S cluster assembly [48,49,50]. These data suggest that the dysregulation of iron may cause ferroptotic cell death, which warrants further study. Notably, the inhibition of peroxynitrite formation by iNOS inhibitors or superoxide scavengers prevents β-cell destruction and diabetes development in non-obese diabetic NOD mice [51,52].

### 4.2. The Role of NADPH Oxidase in Ferroptotic Signaling

To learn more about the characteristics of the effector that mediates iron-induced cell damage, the effects of the NADPH oxidase enzyme system are emphasized below. Emerging evidence suggests that NADPH oxidase (NOX) is a major source of extra-mitochondrial superoxide radicals in β-cells [53,54,55,56,57]. The enzyme NADPH oxidase is a multi-subunit enzyme, and the assembly of the active enzyme complex is described in Ref. [57]. Broniowska et al. demonstrated that peroxynitrite formation by cytokines was reduced in the absence of superoxide, which suggests that NOX is involved in iron-mediated pancreatic β-cell damage [58]. Moreover, it should be noted that the activation of mitochondrial H202 and hydroxyl radical formation contribute to cytokine-induced pancreatic β-cell cytotoxicity [59]. However, evidence suggests that iron-induced NF-kB mediated NOX expression exerts inflammatory effects in atherosclerosis [60]. The precise connection between NOX and iron-induced β-cell inflammation is yet to be fully understood. In addition, activation of JNK signaling occurs downstream of iron- and NOX-induced β-cell apoptosis [33,61]. Even though JNK has many downstream targets, p66Shc, a 66 kDa Src collagen homolog (Shc) adaptor protein, was found to link mitochondrial ROS production in pancreatic β-cells in response to JNK activation under lipotoxic conditions [62,63,64,65]. Phosphorylation of p66Shc at Ser36 triggers its mitochondrial localization, where it generates H_2_O_2_ via its oxidoreductase activity [66,67,68]. Additionally, under glucolipotoxic conditions, elevated levels of LIPs by DMT1 mediate mitochondrial dysfunction and β-cell destruction [69], suggesting the possibility of p66Shc activation. However, further studies are required. In contrast, angiotensin II- (ANG-II)-induced NOX activation increased the LIP and iron-dependent oxidative stress by JNK-p66Shc mediated ferritin degradation in human umbilical vein endothelial cells and HT22 neuronal cells [70]. Interestingly, chronic hyperglycemia and ANG-II type 1 receptor-induced pro-inflammatory cytokine secretion in human islets cause superoxide production and p47^phox^ and p22^phox^ expression, which impairs insulin secretion and inflammation. However, inhibition of the ANG-II II type 1 receptor downregulates NADPH oxidase, which in turn suppresses oxidative stress, thus improving β-cell insulin secretion and decreasing β-cell inflammation [71,72,73]. These findings also provide a potential mechanism for how NOX-dependent H_2_O_2_ production is a likely cause of glucose and ANG-II working together to induce LIP and impairment in insulin secretion and the induction of β-cell dysfunction. Therefore, further research into this connection should provide insightful information regarding β-cell dysfunction during diabetes. 

Indeed, Weaver et al. demonstrated that the pro-inflammatory cytokines induced 12-lipoxygenase expression and increased the flux of hydroxyeicosatetraenoic acid (12-HETE) from arachidonic acid (AA), impairing β-cell function by NOX-1 induction [74,75]. The lipoxygenases (LOXs) are non-heme iron-containing dioxygenases that catalyze the formation of a complex array of bioactive LOOHs that regulate cell signaling. Moreover, children newly diagnosed with type 1 diabetes have very high LOX-induced HETE plasma concentrations [76]. However, it is unknown how and to what extent HETE contributes to the pathophysiology of pancreatic β-cells in diabetes. The ability of LOX-overexpressing cells to undergo ferroptosis may be attributed to an initial increase in the concentration of a particular LOOH, which can later break down to produce alkoxyl and/or hydroxyl radicals via the Fenton reaction for nonenzymatic lipid peroxidation [77,78,79]. Moreover, to specifically initiate the production of the ferroptotic hydroperoxy-phospholipid hydroperoxyeicosaetetranoic acid, LOX induces circumstances in which PEBP1, a Raf-1 kinase inhibitory protein, eagerly binds LOX, altering its catalytic competence from free AA to AA-PE [80,81]. Interestingly, PEBP1 expression is high in the pancreatic islets, and the deletion of PEBP1 significantly suppressed streptozotocin-induced activation of β-cell destruction and increased β-cell mass [82]. Hence, it is tempting to speculate that PEBP1 is involved in ferroptosis signaling-induced β-cell dysfunction, which warrants further investigation.

### 4.3. ACSL4 in β-Cell Ferroptosis

Moreover, lipid peroxidation in ferroptosis is supported by acyl-CoA synthetase long-chain family member 4 (ACSL4), an acyl CoA synthetase enzyme that acylates PUFA and generates fatty acyl-CoA esters, which are transesterified into phospholipids [83]. Notably, ACSL4 is present in insulin-secretory granules and is involved in insulin secretion [84]. In addition, ACSL4 is essential for the induction of lipid oxidation during ferroptosis [85]. However, the relevance of ACSL4 in β-cell ferroptosis remains unexplored and enigmatic. Together, these findings suggest that the dysregulation of iron handling and lipid peroxidation disrupts the cellular redox balance in organelles that orchestrate ferroptotic death signals.

### 4.4. Glutathione System in β-Cell Ferroptosis

To control the intracellular redox balance, cells have evolved a network of antioxidant systems to scavenge ROS, among which the glutathione (GSH)-dependent system may be particularly important. However, due to low levels of protective antioxidant enzymes compared with that in other tissues, redox imbalance is apparently a significant hallmark of pancreatic β-cell malfunction and death [86]. It is plausible that the ferroptotic effects of the diabetic milieu (glucose, cytokines, and fatty acids) may be mediated, in part, through the inhibition of the GSH system and subsequent activation of ROS production. However, under conditions of oxidative stress, the redox status of cells results in the loss of GSH, which lowers their reducing ability and can only be restored by producing fresh GSH [87]. Therefore, the GSH/GSSG ratio can be used as a sign of the redox environment inside the cell. Glutathione peroxidase 4 (GPX4) is the only enzyme that lowers lipid hydroperoxides to match alcohols or water by reducing free hydrogen peroxide [88]. Mechanistically, GSH synthesis is required for GPX4 activity, which offers reducing equivalents to eliminate oxidative species, supported by the fact that mice lacking the GSH-synthesizing enzyme glutamylcysteine synthetase and GPX4 die at the same developmental stage [89,90]. Moreover, GSH synthesis is manipulated by the availability of cysteine, and its uptake relies on the glutamate/cysteine antiporter (system xCT), which is composed of the transmembrane protein transporter SLC7A11 and the transmembrane regulatory protein SLC3A2 [91,92,93,94]. However, new experimental evidence demonstrates that the pharmacological inhibition of system xCT by erastin or GPX4 inactivation by RSL3 induces ferroptotic cell death in human islets. In addition, ferrostatin-1 (a ferroptosis inhibitor) or desferrioxamine, an iron chelator, prevents ferroptotic death and improves the function of human islets [95]. Further studies have shown that GPX4 overexpression prevents the accumulation of phospholipid hydroperoxides that make pancreatic β-cells susceptible to ferroptotic-like cell death by free fatty acids [96]. Supporting this, Krümmel et al. found that the overexpression of GPX4 efficiently prevents tert-butyl hydroperoxide and pro-inflammatory cytokine-induced lipid peroxidation and ferroptotic β-cell death [97]. Importantly, it has been shown that the availability of reduced GSH is regulated by NADPH supply, which is utilized by GSH reductase [98]. The major sources of NADPH are glucose-6-phosphate dehydrogenase and 6-phosphoglucanate dehydrogenase of the pentose phosphate pathway enzymes [99,100,101]. Importantly, glucose-6-phosphate dehydrogenase expression and activity are decreased by hyperglycemia, with a reduction in GSH reductase activity, rendering pancreatic β-cells susceptible to oxidative damage via the GSH/GSSH ratio [102,103]. Additionally, glutamine provides precursors for GSH production, resulting in a decrease in the steady-state level of lipid oxidation products, a crucial component of cell viability [104,105]. In this context, glutamine availability is sensed by Glutaminase 1 (GLS1), which converts glutamine into glutamate for GSH synthesis and plays an important role in insulin secretion [106,107,108,109]. Notably, we discovered that endogenous GLS1 mRNA and protein expression were suppressed upon exposure to diabetic milieu conditions (hyperglycemia, streptozotocin, and H_2_O_2_), leading to a reduction in GSH synthesis. This correlates with a significant decline in the GSH/GSSG ratio associated with the accelerated degradation of xCT and GPX4. In particular, a drop in GPX4 levels may cause phospholipid hydroperoxides to accumulate, which makes pancreatic cells more prone to cell death that resembles ferroptosis [110,111]. These results contribute to the integration of intracellular processes with an increase in ROS levels caused by diabetic milieu conditions (hyperglycemia, streptozotocin, and H_2_O_2_), resulting in the onset of islet dysfunction and diabetes. Further investigation is required to interpret these findings.

## 5. Therapeutic Agents Targeting Inhibition of Ferroptotic-Death

**Figure 2:**
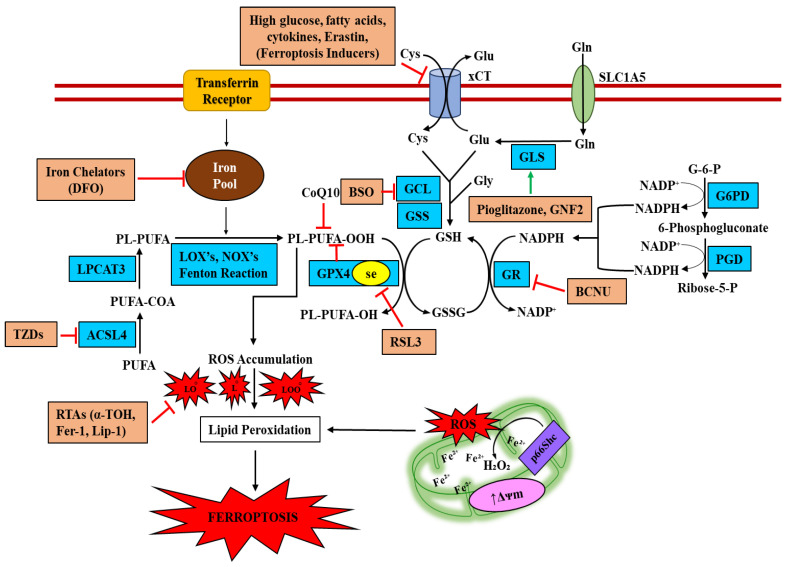
Role of iron in β-cell ferroptotic signaling and therapeutic agents targeting the inhibition of ferroptotic-death.

Notably, evidence indicates that iron accumulation and lipid peroxidation are associated with ferroptotic cell dysfunction. Therefore, medications that lower iron accumulation or lipid peroxidation inhibitors are helpful in treating diabetes, obesity, and peripheral insulin resistance. Iron-chelating substances are frequently used in the clinical context because they can easily limit and redistribute systemic iron. A growing body of evidence suggests that the chelators deferoxamine and deferiprone ameliorate experimental diabetes and preserve β-cell mass, protecting β-cells from apoptosis [33,112,113,114,115,116,117]. In contrast, the metabolic response to iron overload is tightly regulated by DMT1, and inhibition of DMT1 or iron restriction improved the glucose tolerance and circulating insulin levels in high-fat diet-induced diabetes and multiple low-dose streptozotocin-induced islet inflammation [7,17,33,69,118]. However, a number of small-molecule DMT1-mediated iron transport inhibitors have been studied. For example, ferristatin II (NSC306711) attenuates DMT1-mediated iron uptake and induces transferrin receptor degradation, which inversely correlates with the expression of lipid peroxidative genes and proteins to restrain ferroptosis [119,120,121,122]. To date, the beneficial activities of ferristatin II have not been studied in iron-related metabolic diseases and require further investigation. The antioxidant activity of the selenium-containing drug ebselen potently suppresses DMT1-mediated iron absorption and reduces iron-induced ROS production in Alzheimer’s disease [123,124]. Further studies suggest that ebselen ameliorates lipotoxic dysfunction by inhibiting oxidative stress and preserving insulin secretion and β-cell mass in Zucker diabetic models, as well as in other experimental diabetes models [125,126,127]. Additionally, pioglitazone, a member of the thiazolidinedione class of anti-diabetic medications, binds to and stabilizes mitoNEET, an inhibitor of the mitochondrial iron uptake protein, thereby inhibiting mitochondrial labile iron accumulation and reducing iron-mediated ROS formation [128,129,130,131,132]. Additionally, we showed that pioglitazone treatment reduces hyperglycemia-induced β-cell oxidative stress by boosting GLS1 stability and activity. Further research demonstrated that pioglitazone treatment restores both the GSH/GSSG ratio and GPX4 protein levels under hyperglycemic conditions, demonstrating that the protective effect of pioglitazone on β-cell apoptosis is dependent on antioxidants and inhibitors of ferroptosis [110]. Additionally, pioglitazone inhibits the expression of COX-2, which is stimulated by traumatic brain injury, most likely by interfering with the process of reducing ROS formation by blocking neuronal ferroptosis [133]. Additionally, pioglitazone has been shown to inhibit ACSL4, which is required for the execution of ferroptosis, and hence decreases mouse embryonic fibroblast ferroptosis [85]. The fundamental processes that initiate this paradoxical occurrence of ACSL4 inhibition and suppression of ferroptosis to enhance metabolic health are not entirely understood. Additionally, coenzyme Q, vitamin E, and di/tetrahydrobiopterin have shown promise as new therapeutic approaches to disease in investigations of the function of these endogenous antioxidants in ferroptosis inhibition [134,135,136,137]. Interestingly, c-Abl, which is elevated by metabolic stress in β-cells, accelerates lipid peroxidation and ferroptosis induced by GPX4 degradation by GLS1 inhibition. Additionally, the blockage of c-Abl by GNF2 allows cells to use glutamine metabolism (glutaminolysis) to produce GSH for β-cell survival and growth [111]. However, it is widely known that LOX inhibitors may inhibit the majority of ferroptotic cell deaths by preventing mitochondrial malfunction [138,139,140,141,142]. Furthermore, GLP-1 receptor agonist (GLP-1-RA) therapy and/or iron chelation enhances mitochondrial performance and restores β-cell function. In Wolfram syndrome and other types of diabetes linked to iron dysregulation, treatment with GLP-1-RA, likely enhanced by iron chelation, should be considered [143,144]. In general, it has become obvious that targeting iron metabolism and ferroptosis offers a compelling new therapeutic strategy for many disorders because of the significant gains in our understanding of the role of iron and ferroptotic damage in a variety of diseases (Figure 2).

## 6. Conclusions

According to experimental data, iron metabolism plays a role in the malfunction of pancreatic β-cells during the development of diabetes. The management of β-cell failure and T2D may be greatly affected by an understanding of this complex scenario and the role of iron-activated ferroptosis redox-regulated pathways. It is almost certain that these efforts will be helpful in the search for novel and efficient treatments for diseases associated with abnormal iron metabolism, even though more research is still required to thoroughly examine the illnesses linked to abnormal iron metabolism in pancreatic β-cells.

## Data Availability

Not applicable.

## Abbreviations

DMT1	Divalent metal transporter 1
FPN	Ferroportin
TrfR	Transferrin receptor
STZ	Streptozotocin
c-Abl	c-Abelson tyrosine kinase
LOX	Lipoxygenase
LCN2	Lipocalin-2
ACSL4	Acyl-CoA Synthetase Long Chain Family Member 4
HFD	High fat diet; NO—Nitric oxide; GLS1—Glutaminase 1
